# Quantitative total serum IgG4 reporting in human paragonimiasis: a scoping review

**DOI:** 10.1186/s41182-026-01080-9

**Published:** 2026-09-21

**Authors:** Hidenori Takahashi, Takumi Yasuda, Yugo Satake, Hiroki Nagamatsu, Ryutaro Hirose, Mio Toyama-Kosaka, Shinichiro Ota, Miwa Morikawa, Mio Kokubo-Tanaka, Masaharu Shinkai

**Affiliations:** 1 Department of Respiratory Medicine, Tokyo Shinagawa Hospital, 6-3-22, Higashi-Oi Shinagawa-Ku, Tokyo, 140-8522 Japan; 2 Division of Parasitology, Department of Infectious Diseases, Faculty of Medicine, University of Miyazaki, 5200 Kiyotakecho Kihara, Miyazaki City, Miyazaki 889-1692 Japan

**Keywords:** Paragonimiasis, IgG4-related disease, Serum IgG4, Scoping review, Differential diagnosis

## Abstract

**Background:**

Paragonimiasis is a food-borne parasitic disease caused by lung flukes belonging to the genus *Paragonimus*. It can produce pulmonary, pleural, and other organ manifestations that overlap clinically, radiologically, and histopathologically with malignancy, tuberculosis, and immune-mediated disorders. Elevated total serum immunoglobulin G4 (IgG4) concentrations and IgG4-positive plasma cell infiltration may mimic IgG4-related disease (IgG4-RD), but quantitative serum IgG4 levels in paragonimiasis have not been systematically characterized.

**Methods:**

We conducted a multi-database scoping review of human paragonimiasis case reports and case series published from January 1, 1965, to December 31, 2025. PubMed, Ichushi-Web, Chinese National Knowledge Infrastructure (CNKI), and KoreaMed were searched using database-specific strategies, supplemented by multilingual Google Scholar checks. The review was reported in accordance with the PRISMA-ScR checklist.

**Results:**

Among 610 reports comprising 886 cases, quantitative serum IgG4 values were reported in 10 reports (1.6%) and 12 cases (1.4%). The countries of first-author affiliation were Japan (*n* = 6), China (*n* = 2), Thailand (*n* = 1), and France (*n* = 1), and all reports were published in 2012 or later. The 12 cases included pulmonary, pleural/intrathoracic, pericardial, central nervous system, and adrenal presentations. Among the 11 cases reported in mass concentration units, serum IgG4 values ranged from 94.1 to 3,350 mg/dL, and 10 values were elevated. The remaining case was reported as elevated (1,990 IU/mL) but was not converted to mass concentration units. Tissue pathology was described in 8 cases, and IgG4-positive plasma cell infiltration, IgG4-positive inflammation, or an increased tissue IgG4/IgG ratio was reported in 4 cases. None of the pathology reports described storiform fibrosis or obliterative phlebitis, which are hallmarks of IgG4-RD. Based on the information available in the original publications, none of the 12 cases were mapped as probable or definite IgG4-RD.

**Conclusion:**

Quantitative serum IgG4 values are rarely reported in paragonimiasis case literature, and selective testing and reporting precluded estimation of the prevalence of serum IgG4 elevation. Serum IgG4 elevation and IgG4-positive plasma cells should be interpreted together with exposure history, parasite-directed evidence, and histopathology. Early consideration of paragonimiasis may prevent delayed diagnosis and inappropriate immunosuppression owing to misdiagnosis.

**Supplementary Information:**

The online version contains supplementary material available at https://doi.org/10.1186/s41182-026-01080-9.

## Background

Paragonimiasis is a food-borne parasitic disease caused by lung flukes belonging to the genus *Paragonimus*. It can have diverse effects on the lungs including eosinophil-predominant granulomatous inflammation related to parasite migration and egg deposition, often with hemorrhage, necrosis, and pleuritis. The manifestations include pulmonary masses or nodules, cavitation, pleural effusion or thickening, and pneumothorax [1, 2]. Consequently, paragonimiasis often requires differentiation from lung cancer [3], tuberculosis [4], and inflammatory disorders such as eosinophilic pneumonia [5].

IgG4-related disease (IgG4-RD) is a systemic fibroinflammatory disorder that classically involves the pancreatobiliary system and salivary glands but can also present with thoracic manifestations such as pulmonary nodules/masses, interstitial lung abnormalities, pleural thickening, and pleural effusion [6, 7]. Elevated serum IgG4 levels and lymphoplasmacytic infiltration with increased IgG4-positive plasma cells are used to make the diagnosis. However, these findings are not specific to IgG4-RD and can also occur in infections and other chronic inflammatory conditions, and overlapping clinical, radiologic, and pathologic features can make diagnosis difficult [6–8]. The clinical manifestations of paragonimiasis and IgG4-RD overlap, and paragonimiasis can be difficult to distinguish from IgG4-RD if the total serum IgG4 level is elevated or IgG4-positive plasma cells are present on histology [9, 10]. If paragonimiasis is misdiagnosed as IgG4-RD or another immune-mediated disorder, this may lead to the initiation of inappropriate immunosuppressive treatment [10, 11].

However, how often quantitative total serum IgG4 is reported in paragonimiasis, and the clinical and pathologic contexts in which it is evaluated, have not been systematically assessed. The potential for misdiagnosis of paragonimiasis as IgG4-RD is thus unclear. Accordingly, we conducted a scoping review to: (1) quantify how often quantitative total serum IgG4 is reported in the paragonimiasis case literature; (2) describe the clinical, imaging, and pathologic features and reported *Paragonimus* species of cases with quantitative total serum IgG4 values; and (3) describe the clinical and pathologic contexts in which IgG4-RD may be considered in the differential diagnosis of paragonimiasis, based on a review of the literature.

## Methods

### Design

We conducted a scoping review and reported the results in accordance with the PRISMA-ScR checklist [12].

### Data sources and search strategy

We conducted a multi-database scoping review of human paragonimiasis case reports and case series. The search covered reports published from January 1, 1965, to December 31, 2025. The 1965 start date was selected pragmatically to coincide with the publication of seminal methods for quantitative serum immunoglobulin measurement [13, 14]. We searched PubMed, Ichushi-Web, Chinese National Knowledge Infrastructure (CNKI), and KoreaMed databases using database-specific search strategies. Ichushi-Web, CNKI, and KoreaMed were selected to improve coverage of Japanese-, Chinese-, and Korean-language case reports that may not be indexed in PubMed. For each database, we used both broad-based paragonimiasis case report/case series and IgG4/IgG4-related disease-focused search strategies. No language restrictions were applied.

For Ichushi-Web, CNKI, and KoreaMed, language-specific search terms were used to capture Japanese-, Chinese-, and Korean-language reports, respectively. The full search strategies, search dates, and database-specific search results are provided in Additional file 1, Table S1.

### Supplementary Google Scholar checks

To reduce the risk of missing reports not captured by the database searches, supplementary Google Scholar checks were performed in English, Japanese, Chinese, and Korean. For the English-language Google Scholar search, the first 300 ranked results were screened. For the Japanese-, Chinese-, and Korean-language Google Scholar searches, up to the first 50 ranked results were screened, except when fewer than 50 results were returned.

Records already identified through PubMed, Ichushi-Web, CNKI, or KoreaMed were logged as source overlap and were not counted as additional records in the PRISMA flow diagram. Only additional unique records not captured by the database searches were entered into the “other methods” arm of the flow diagram.

### Eligibility criteria

Case reports and case series on paragonimiasis were included. Reports were eligible for inclusion if they described one or more human cases of paragonimiasis and provided individual case-level clinical information. Reports were excluded if they did not describe human paragonimiasis, did not provide individual case-level data, were reviews or diagnostic studies without extractable clinical cases, described only animal or basic research, or represented duplicate or overlapping publications of the same case(s).

### Record management and study selection

Search results were first managed separately within each source. Within-source duplicate records, including duplicates between the broad-based paragonimiasis and IgG4-focused searches, were removed before source-specific title/abstract screening. Because bibliographic metadata, titles, and indexing formats differed between the English-, Japanese-, Chinese-, and Korean-language databases, exhaustive cross-database deduplication was not performed at this stage. Instead, each source-derived record set was screened independently according to the eligibility criteria.

Records retained after source-specific screening were then pooled for cross-source adjudication. The pooled records were assessed for cross-source duplicate records and duplicate or overlapping publications using PubMed ID (PMID), Digital Object Identifier (DOI), title, authors, journal, year of publication, language, and other available bibliographic information. Reports identified as the same bibliographic publication across databases were counted only once. Cross-source duplicate records were defined as the same bibliographic report identified in more than one database and were distinguished from duplicate or overlapping publications, which were separate reports describing the same patient(s) or an overlapping case series.

Reports retained after cross-source adjudication were sought for retrieval. Reports for which neither full text nor sufficient clinical information could be obtained were classified as reports not retrieved. For CNKI-indexed reports without full text accessible through institutional subscription access, eligibility assessment was based on the bibliographic record and abstract when these provided sufficient individual case-level information.

**Fig. 1:**
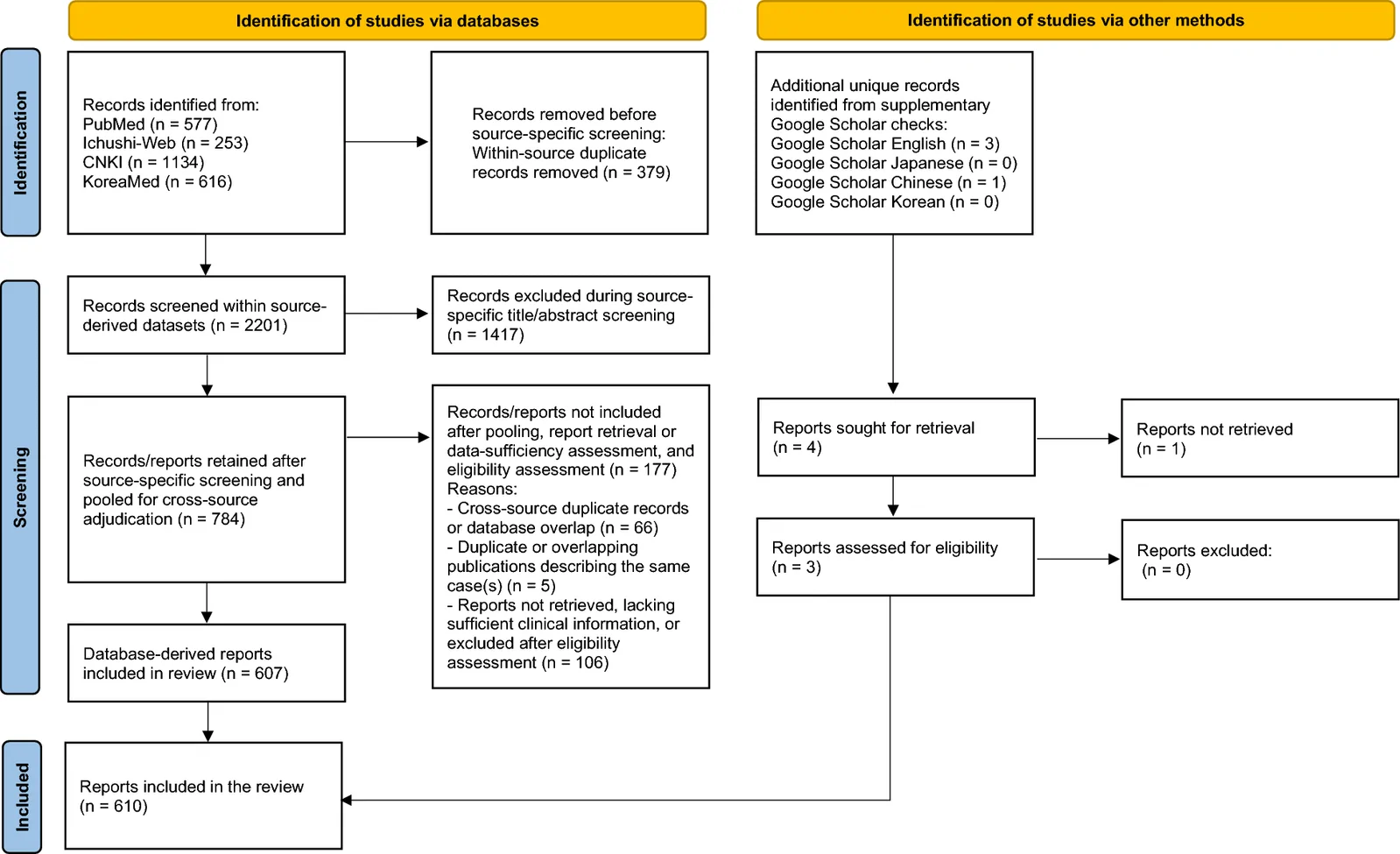
Modified PRISMA-ScR flow diagram of report selection. Database searches identified 2,580 records from PubMed (*n* = 577), Ichushi-Web (*n* = 253), CNKI (*n* = 1,134), and KoreaMed (*n* = 616). After removal of 379 within-source duplicates, 2,201 records were screened, of which 1,417 were excluded during source-specific title/abstract screening. The remaining 784 records/reports underwent cross-source adjudication. **Sixty-six** cross-source duplicate records, **5** duplicate or overlapping publications describing the same patient(s) or overlapping case series, and 106 records/reports that were not retrieved, lacked sufficient clinical information, or were excluded after eligibility assessment were not included, yielding 607 database-derived reports. Supplementary multilingual Google Scholar checks identified four additional unique records not captured by the database searches; one was not retrieved and three were included. Overall, 610 reports comprising 886 cases were included in the review. IgG4-RD, IgG4-related disease; PRISMA-ScR, Preferred Reporting Items for Systematic Reviews and Meta-Analyses Extension for Scoping Reviews

Retrieved reports were assessed for eligibility, and separate publications confirmed to describe the same patient or overlapping case(s) were excluded as duplicate or overlapping case publications at this stage. The study selection process is summarized in a modified PRISMA-ScR flow diagram (Fig. 1).

**Alt text (Figure 1):** Modified PRISMA-ScR flow diagram showing the identification and selection of human paragonimiasis case reports and case series from PubMed, Ichushi-Web, CNKI, KoreaMed, and supplementary multilingual Google Scholar checks. The diagram shows 2,580 database records identified, 379 within-source duplicates removed, 2,201 records screened, 784 records/reports undergoing cross-source adjudication, 607 database-derived reports included, three additional reports included through Google Scholar checks, and a final total of 610 reports comprising 886 cases.

### Data extraction

For each included report, we extracted the publication year, first author, first-author country based on affiliation, number of cases, *Paragonimus* species (if reported), primary site of infection, and whether quantitative serum IgG4 values were provided. For reports that included quantitative serum IgG4 values, we also extracted data on the reported clinical presentation, laboratory findings, imaging findings, pathological findings, basis for diagnosing paragonimiasis, initial diagnosis, and the differential diagnosis. Serum IgG4 values were extracted in the units originally reported. Values in g/L or mg/mL were converted to mg/dL for descriptive comparison, whereas values in IU/mL were retained without conversion.

For CNKI reports assessed using abstract-level information only, only those variables explicitly described in the bibliographic record or abstract were extracted.

### IgG4-RD criteria mapping

To contextualize diagnostic overlap between paragonimiasis and IgG4-RD, we assessed whether each case with a quantitative serum IgG4 result met the 2020 revised comprehensive diagnostic criteria for IgG4-RD [6] using only the information available in the publication (Additional file 2, Table S2). Cases were mapped as possible, probable, or definite IgG4-RD when the published information fulfilled the corresponding criteria. This mapping was conducted to characterize how each case might be interpreted within an IgG4-RD diagnostic framework based on published information alone. It was not intended to adjudicate IgG4-RD as the final diagnosis and did not incorporate the final diagnosis of paragonimiasis into the classification.

### Analysis

Data were managed in Microsoft Excel. Descriptive analyses comprised frequency counts for categorical variables and ranges for continuous variables.

## Results

The database searches identified records from PubMed (*n* = 577), Ichushi-Web (*n* = 253), CNKI (*n* = 1,134), and KoreaMed (*n* = 616). After removing within-source duplicate records (*n* = 379), 2,201 records were screened within source-derived datasets. Of these, 1,417 records were excluded during source-specific title/abstract screening, and 784 records/reports were retained for cross-source adjudication.

Among the 784 records/reports pooled for cross-source adjudication, 177 were not included: 66 were cross-source duplicate records identified in more than one database, 5 were duplicate or overlapping publications describing the same patient(s) or overlapping case series (Additional file 3, Table S3), and 106 were not retrieved, lacked sufficient clinical information for eligibility assessment, or were excluded after eligibility assessment. This process yielded 607 database-derived reports for inclusion.

Supplementary Google Scholar checks identified 4 additional unique records not captured by the database searches, of which 3 were published in English and 1 in Chinese. Of these 4 records, 1 was not retrieved, whereas the remaining 3 were assessed for eligibility and included. In total, 610 reports, comprising 886 cases, were included in the review (Fig. 1).

The characteristics of the reports are summarized in Table 1, and a complete list of reports is provided in Additional file 4, Table S4. Among the reports, the most common country of first-author affiliation was China (216 reports), followed by Japan (211 reports), Korea (91 reports), and the United States (27 reports) (Table 1). Reports with pleuropulmonary primary sites were more frequent than those with other primary sites (398/610 reports, 65.2%, vs 212/610 reports, 34.8%). Species-level identification was reported in 278 of the 610 reports (45.6%).

**Table 1:** Characteristics of included reports

	Overall	China	Japan	Korea	USA	Thailand	France	India	Other
Reports, *n*	610	216	211	91	27	12	9	8	36
Primary site, pleuropulmonary/other (reports), *n*	398/212	123/93	164/47	41/50	22/5	9/3	6/3	7/1	26/10
Species specified, *n*	278	22	171	50	18	5	2	1	9
Reported species	–	Pw; Pskr; Phet	Pw; Pmiy; Phet	Pw	Pw; Pk; Pmex	Phet; Pphet; Pw	Pw	Pw	Pw; Put; Pmex
Quantitative serum IgG4 reported, *n*	10	2	6	0	0	1	1	0	0

Characteristics of the 610 included human paragonimiasis case reports and case series published from 1965 to 2025, summarized by country of first-author affiliation, primary-site category (pleuropulmonary vs other), *Paragonimus* species, and quantitative serum IgG4 reporting

Country was defined by first-author affiliation

Pw, *Paragonimus westermani*; Pmiy, *Paragonimus miyazakii*; Pskr, *Paragonimus skrjabini*; Pk, *Paragonimus kellicotti*; Pmex, *Paragonimus mexicanus*; Put, *Paragonimus uterobilateralis*; Phet, *Paragonimus heterotremus*; Pphet, *Paragonimus pseudoheterotremus*

Quantitative serum IgG4 values were reported in only 10 of the 610 reports (1.6%), and 12 of the 886 cases (1.4%) reviewed [9–11, 15–21]. All 10 reports were published in 2012 or later and the countries of first-author affiliation were Japan (*n* = 6), China (*n* = 2), Thailand (*n* = 1), and France (*n* = 1). The 12 cases included children and adults of both sexes. Primary sites were the lung (7 cases), pleura/intrathoracic compartment (2 cases), central nervous system (1 case), cardiac/pericardial compartment (1 case), and adrenal gland (1 case). The radiologic findings included nodular or consolidative lung lesions, cavitary lesions, pleural effusion, pleural thickening, pericardial effusion, pneumothorax, and hepatic or adrenal lesions. Reported initial or differential diagnoses included tuberculosis, IgG4-RD, eosinophilic granulomatosis with polyangiitis, hematologic disease, and lung carcinoma. The degree of IgG4-RD diagnostic relevance varied among the 12 cases: IgG4-RD was explicitly considered or not excluded in 8 cases, discussed as a diagnostic issue at the report level in 3 cases, and was not a central diagnostic consideration in 1 case (Table 2).

**Table 2:** Case-level characteristics of paragonimiasis with reported serum IgG4 measurements

No.	Author, year	Country; age/sex	Primary site	Key imaging findings	Other initial/differential diagnoses	Key laboratory findings	IgG4-RD consideration in original report	Pathology	Species and Paragonimus diagnostic basis	2020 RCD mapping	Treatment/IgG4 course
1	Torigoe [19], 2012	Japan; 44/F	Lung	LUL patchy opacity evolving to a cavitary nodule; migratory opacity	Autoinflammatory syndrome	AEC 372/μL; IgG 1,686 mg/dL; IgG4 190 mg/dL	Considered as IgG4-related lung disease	TBLB negative for parasites or IgG4-RD; BAL eosinophilia	Pw; antibody by multiple-dot and microtiter-plate ELISA	Possible only	Praziquantel only; follow-up NR
2	Saeki [9], 2015 case 1	Japan; 49/M	Lung	RUL consolidation followed by a right middle-lobe nodule	Not stated	AEC 82/μL; IgG NR; IgG4 201 mg/dL	Discussed as a differential	Ova with IgG4-positive plasma cell infiltration	Pw; biopsy + antibody ELISA	Possible only	Praziquantel only; normalized by 4 months
3	Saeki [9], 2015 case 2	Japan; 56/F	Lung	LUL and lower-lobe cavitary lesions	Not stated	AEC 229/μL; IgG NR; IgG4 374 mg/dL	Discussed as a differential	Dense plasmacytic infiltration; majority IgG4-positive	Pw; antibody ELISA	Possible only	Praziquantel only; decreased to near-normal by 11 months
4	Roque [17], 2019	France; 32/M	Lung	Migratory opacities, LUL cavity, pulmonary nodules, and bilateral pleural effusions	Pneumonia; tuberculosis/NTM; malignancy/lymphoma; EGPA/GPA; fungal or parasitic infection	AEC 650 → 2,000–4,000/μL; IgG NR; IgG4 1,100 mg/dL	Explicitly considered; marked eosinophilia argued against it	BAL eosinophilic alveolitis with Charcot–Leyden crystals; TBLB nonspecific lymphoplasmacytic infiltration	Paragonimus sp.; eggs in sputum and stool	Possible only	Praziquantel only; follow-up NR
5	Jaroensukrungruang [16], 2020	Thailand; 58/F	Adrenal	Multifocal hepatic lesions followed by a new left adrenal rim-enhancing lesion	Liver abscess; parasitic infection; amoebic abscess	AEC 2,100/μL; IgG 4,240 mg/dL; IgG4 3,350 mg/dL	Initially diagnosed and treated as IgG4-RD; later attributed to paragonimiasis	Liver: eosinophilic abscess and Splendore–Hoeppli phenomenon, IgG4/IgG ratio 80%; adrenal: granulomatous inflammation with Paragonimus eggs	Pw; eggs in adrenal tissue and stool + serology	Possible only	Immunosuppression followed by praziquantel and withdrawal of immunosuppression; eventually normalized
6	Akaike [15], 2022	Japan; 59/M	Pleural/intrathoracic	Posterior apical mass with arterial incorporation on CT; MRI showed low T1, heterogeneous high T2, and enhancement	Posterior mediastinal tumor, especially neurogenic tumor	AEC 480/μL; IgG 1,218 mg/dL; IgG4 94.1 mg/dL	Not considered	Xanthogranulomatous inflammatory tissue containing parasite eggs; no tissue eosinophils described	Pw; eggs in CT-guided biopsy and high *P. westermani* antibody titer by microplate ELISA	Did not meet possible criteria	Praziquantel only; follow-up NR
7	Yamamuro [11], 2022	Japan; 48/F	Central nervous system	Multiple pulmonary nodules and intracranial hemorrhagic/ring-enhancing lesions	EGPA	AEC 5,170/μL; IgG NR; IgG4 1,990 IU/mL	Explicitly considered	Trematode eggs in brain tissue; inflammatory infiltrate mainly lymphocytes and plasma cells	Pw; brain-tissue eggs + positive serum testing for *P. westermani*	Possible only	High-dose glucocorticoids followed by praziquantel; follow-up NR
8	Taku [18], 2024	Japan; 12/M	Pleural/intrathoracic	Bilateral pleural effusions with pleural thickening	Not stated	AEC 650/μL; IgG NR; IgG4 1,350 mg/dL	Assessed; characteristic features reportedly absent	No biopsy	Pmiy; antibody ELISA	Possible only	Praziquantel only; progressively normalized
9	Zhang [21], 2025	China; 10/M	Cardiac/pericardial	Persistent pericardial and pleural effusions	Paragonimiasis	AEC 2,030 → 480/μL; IgG 1,910 → 1,470 mg/dL; IgG4 1,100 mg/dL	Considered and not excluded	No biopsy	Pw; IgG serology (method NR)	Possible only	Multiple praziquantel courses, then methylprednisolone; decreased but remained elevated
10	Takahashi [10], 2025	Japan; 50/M	Lung	Bilateral upper-lobe irregular nodular/ground-glass lesions	Lung carcinoma	AEC 1,580/μL; IgG 1,375 IU/mL; IgG4 491 mg/dL	Initially diagnosed and treated as IgG4-RD	IgG4-positive lymphoplasmacytic and eosinophilic inflammation; retrospective review identified parasite eggs	Pw; antibody ELISA + retrospective histology	Possible only	Prednisolone followed by praziquantel; little change with steroids, rapid normalization after praziquantel
11	Yin [20], 2025 case 1	China; 40 s/F	Lung	RML lesion with large right pleural effusion	Tuberculosis	AEC 1,610/μL; IgG 2,080 mg/dL; IgG4 1,133 mg/dL	Included in the differential	No biopsy	Species undetermined; antibody ELISA	Possible only	Praziquantel; decreased toward normal
12	Yin [20], 2025 case 2	China; 60 s/F	Lung	Multiple cavitary lesions and bilateral hydropneumothorax	Leukemia; pulmonary infection; eosinophilia	AEC 12,860/μL; IgG 3,100 mg/dL; IgG4 2,410 mg/dL	Diagnostic overlap discussed; case-specific consideration unclear	No biopsy	Species undetermined; antibody ELISA	Possible only	Prednisone before diagnosis, followed by praziquantel; decreased but remained elevated

Case-level summary of 12 paragonimiasis cases from 10 reports providing quantitative total serum IgG4 values, including first-author country, age/sex, primary site, key imaging and laboratory findings, initial or differential diagnoses, IgG4-RD consideration, pathology, *Paragonimus* species and diagnostic basis, 2020 RCD mapping, and treatment/IgG4 course

Serum IgG and IgG4 mass concentrations were converted to mg/dL where possible; values reported in IU/mL were retained in the original units

The 2020 revised comprehensive diagnostic (RCD) criteria were applied retrospectively using only the information reported in each publication. “Possible only” indicates that the case could not be mapped as probable or definite IgG4-RD

*AEC* absolute eosinophil count, *BAL* bronchoalveolar lavage, *CT* computed tomography, *EGPA* eosinophilic granulomatosis with polyangiitis, *ELISA* enzyme-linked immunosorbent assay, *GPA* granulomatosis with polyangiitis, *IgG* immunoglobulin G, *IgG4-RD* immunoglobulin G4-related disease, *LUL* left upper lobe, *MRI* magnetic resonance imaging, *NTM* nontuberculous mycobacteria, *NR* not reported, *Pmiy* *Paragonimus miyazakii*, *Pw* *Paragonimus westermani*, *RCD* revised comprehensive diagnostic, *RML* right middle lobe, *RUL* right upper lobe, *TBLB* transbronchial lung biopsy

Of the 11 cases for which numerical IgG4 values were reported in mass concentration units, the values ranged from 94.1 to 3,350 mg/dL (normal range, 11 to 121 mg/dL). Ten of these 11 cases had elevated concentrations, whereas the remaining case had a value of 94.1 mg/dL. The twelfth case was reported as 1,990 IU/mL and was described as elevated in the original publication. It was retained as reported and was not converted. Serial serum IgG4 results were available in 8 cases. Serum IgG4 decreased after praziquantel without reported glucocorticoid exposure in 4 cases and after praziquantel with prior glucocorticoid exposure in an additional 2 cases. In the remaining 2 cases, immunosuppressive and antiparasitic treatments were administered sequentially or with partial overlap, precluding attribution of the IgG4 decline to a single treatment. The IgG4 levels normalized in 4 of the 8 cases. Reported initial or peak absolute eosinophil counts ranged from 82 to 12,860 cells/µL.

The species was identified in 9 of the 12 cases, of which 8 were identified as *Paragonimus westermani* and 1 as *Paragonimus miyazakii*. Paragonimiasis was diagnosed based on an anti-*Paragonimus* antibody enzyme-linked immunosorbent assay (ELISA) in 8 cases, positive *Paragonimus* immunoglobulin G (IgG) serology without the method stated in 2 cases, and positive serum testing for *P. westermani* in 1 case. Direct detection of *Paragonimus* eggs or egg-like structures in tissue, sputum, stool, or feces was reported in 6 cases. These diagnostic methods were not mutually exclusive.

The tissue pathology was described in 8 cases: lung tissue in 5, posterior mediastinal tissue in 1, brain tissue in 1, and liver and adrenal tissue in 1. IgG4-positive plasma cell infiltration, IgG4-positive inflammation, or an increased tissue IgG4/IgG ratio was reported in 4 of these 8 cases. None of the pathology reports described storiform fibrosis or obliterative phlebitis, the characteristic histological features of IgG4-RD. Based on the 2020 revised comprehensive diagnostic criteria for IgG4-RD, 11 of the 12 cases could be mapped no higher than “possible” IgG4-RD. In the remaining case, the serum IgG4 level was not elevated and IgG4-RD was neither clinically suspected nor supported pathologically, and so did not fulfill the criteria for possible IgG4-RD.

## Discussion

Although the radiologic, serologic, and histologic findings of paragonimiasis and IgG4-RD overlap, this does not imply that the two conditions share a disease mechanism. Among cases with numerically reported serum IgG4 values, the accompanying clinical findings varied and included radiologic abnormalities, eosinophilia, and IgG4-positive plasmacytic infiltration. The relevance to IgG4-RD was therefore graded rather than uniform: Some reports explicitly considered IgG4-RD in the differential diagnosis, whereas others reported serum IgG4 as one component of the overall clinical assessment without making IgG4-RD a central diagnostic consideration. Not all numerically reported serum IgG4 values were elevated. This distinction is important because IgG4-related findings should be treated as prompts to broaden the differential diagnosis, not as pathognomonic of IgG4-RD [6, 8, 22].

Helminth infections can create a type 2/regulatory immune milieu in which chronic parasite antigen exposure may promote an IgG4-skewed humoral immune response [23–26]. Consistent with this biological plausibility, elevated serum IgG4 concentrations and/or IgG4-positive plasmacytic infiltration have been reported in several cases of paragonimiasis [9–11, 16–21]. However, these findings should be interpreted in light of all available evidence and should not be regarded as evidence of IgG4-RD or of a shared fibroinflammatory mechanism between paragonimiasis and IgG4-RD [7, 22]. Although serum IgG4 decreased in temporal association with praziquantel in several cases, prior or concurrent immunosuppressive therapy in some cases precluded attribution of the decline to praziquantel treatment.

Direct parasitological confirmation of paragonimiasis is often difficult because egg shedding is intermittent and tissue sampling may miss egg-containing foci. Microscopic egg-detection rates have been reported to range from 28 to 38% in sputum samples and from 11 to 15% in stool samples, whereas ELISA-based serology has a reported sensitivity of 82–93% and specificity of 98–100% [1]. Assessment of exposure, including ingestion of raw or undercooked freshwater crab or crayfish or meat of paratenic hosts, and residence in, or travel to, endemic areas, remains essential [27, 28]. Because glucocorticoids may lead to transient improvement in inflammatory manifestations, symptomatic response should not be used to exclude parasitic infection [10, 11, 21].

The key distinction between paragonimiasis and IgG4-RD on histopathology is the architecture. In IgG4-RD, increased IgG4-positive plasma cells support the diagnosis only in an appropriate histopathologic context, particularly dense lymphoplasmacytic inflammation with storiform fibrosis and obliterative phlebitis [7, 22]. In paragonimiasis, lesions are typically centered on eggs or migration tracts, with eosinophil-rich granulomatous inflammation, hemorrhage, necrosis, abscess-like change, cavitation, and pleuritis [1, 2, 5, 21]. Repair is therefore more likely to produce encapsulation-type fibrosis or pleural scarring than diffuse fibroinflammatory remodeling. Although chronic paragonimiasis may show fibrosis, pleural thickening, calcification, and other chronic changes, these findings alone do not constitute the histological pattern seen in IgG4-RD [2, 7, 22]. The presence of IgG4-positive plasma cells may raise the possibility of IgG4-RD, whereas the lack of reported storiform fibrosis and obliterative phlebitis limited support for probable or definite IgG4-RD [7, 22].

In this review, the value of the 12 cases with numerically reported serum IgG4 values lies less in estimating the frequency of IgG4 testing than in illustrating the heterogeneous diagnostic contexts in which IgG4 testing occurs. Cases in which IgG4 testing was conducted were characterized by radiologic abnormalities, serosal disease, or mass-forming lesions. Serum IgG4 elevation and/or IgG4-positive plasmacytic infiltration prompted consideration of IgG4-RD in some cases, whereas IgG4-RD was not a central diagnostic consideration in others. Parasite-directed evidence and the available histopathologic findings generally limited support for a diagnosis of probable or definite IgG4-RD.

This review has several limitations. First, although we searched multiple international and regional databases and conducted supplementary multilingual Google Scholar checks, some locally published or non-indexed reports may have been missed. The evidence base was concentrated in East Asia, and so may reflect regional differences in publication and indexing practices as well as the underlying distribution of disease. Second, case reports are subject to selective testing and incomplete reporting. In one report, serum IgG4 was described in qualitative rather than quantitative terms [29], so the report could not be included in the quantitative IgG4 analysis. Diagnostic workups, assay methods, reference ranges, follow-up intervals, and pathology descriptions were not standardized across reports. Consequently, neither the observed frequency of numerical reporting nor the proportion of cases with elevated values could be used to estimate the frequency of IgG4 testing or the prevalence of serum IgG4 elevation in paragonimiasis. Third, country was assigned according to the first author’s affiliation as a pragmatic proxy for the origin of each report. This may not reflect the country in which infection or exposure occurred. Nevertheless, the observed species distribution was broadly compatible with expected geographic patterns [30]. Despite these limitations, as awareness of IgG4-RD and biopsy-driven diagnostic pathways increases, exposure history and parasite-directed testing should be included in the diagnostic evaluation in patients with radiologically prominent presentations, including mass-forming or serosal disease, if IgG4-RD is considered [31].

## Conclusions

Quantitative serum IgG4 values are rarely reported in the paragonimiasis case literature, and selective testing and reporting preclude estimation of the prevalence of serum IgG4 elevation. Although elevated serum IgG4 and IgG4-positive plasma cells sometimes suggested the possibility of IgG4-RD, none of the cases could be mapped as probable or definite IgG4-RD based on the information reported in the original publications. These findings should therefore be interpreted in conjunction with exposure history, parasite-directed evidence, and histopathologic architecture. Early consideration of paragonimiasis may help avoid diagnostic delay and inappropriate immunosuppression.

## Supplementary Information

Additional file 1.

Additional file 2.

Additional file 3.

Additional file 4.

## Data Availability

All data supporting the findings of this study are included within the article and its supplementary information files. The supplementary files provide the full database-specific search strategies and search log, the duplicate or overlapping publication exclusions, the complete line listing of included reports, and the case-level mapping to the 2020 revised comprehensive diagnostic criteria for IgG4-related disease.

## Abbreviations

CT: Computed tomography
ELISA: Enzyme-linked immunosorbent assay
IgG: Immunoglobulin G
IgG4: Immunoglobulin G4
IgG4-RD: Immunoglobulin G4-related disease
PRISMA-ScR: Preferred Reporting Items for Systematic reviews and Meta-Analyses extension for Scoping Reviews

## Declarations

### Use of artificial intelligence tools

During manuscript preparation, a large language model was used to assist with language refinement, organization of text, and preliminary language-assisted reading of selected Chinese- and Korean-language reports. The authors independently reviewed, revised, and verified all extracted data and manuscript content and take full responsibility for the final manuscript.
